# Advanced glycation end-product (AGE)-albumin from activated macrophage is critical in human mesenchymal stem cells survival and post-ischemic reperfusion injury

**DOI:** 10.1038/s41598-017-11773-1

**Published:** 2017-09-14

**Authors:** Myeongjoo Son, Woong Chol Kang, Seyeon Oh, Delger Bayarsaikhan, Hyosang Ahn, Jaesuk Lee, Hyunjin Park, Sojung Lee, Junwon Choi, Hye Sun Lee, Phillip C. Yang, Kyunghee Byun, Bonghee Lee

**Affiliations:** 1 Department of Anatomy & Cell Biology, Graduate School of Medicine, Gachon University, Incheon, 21936 Republic of Korea; 2 Center for Genomics and Proteomics & Stem Cell Core Facility, Lee Gil Ya Cancer and Diabetes Institute, Gachon University, Incheon, 21999 Republic of Korea; 3 Cardiology, Gachon University Gil Medical Center, Incheon, 21565 Republic of Korea; 4 Department of Cardiovascular Medicine, Stanford University, Stanford, CA 94305 USA

## Abstract

Post-ischemic reperfusion injury (PIRI) triggers an intense inflammatory response which is essential for repair but is also implicated in pathogenesis of post-ischemic remodeling in several organs in human. Stem cell therapy has recently emerged as a promising method for treatment of PIRI in human. However, satisfactory results have not been reported due to severe loss of injected stem cells in PIRI including critical limb ischemia (CLI). For investigating the advanced glycation end-product-albumin (AGE-albumin) from activated macrophages is critical in both muscle cell and stem cell death, we evaluated the recovery of PIRI-CLI by injection of human bone marrow derived mesenchymal stem cells (hBD-MSCs) with or without soluble receptor for AGEs (sRAGE). Our results showed that activated M1 macrophages synthesize and secrete AGE-albumin, which induced the skeletal muscle cell death and injected hBD-MSCs in PIRI-CLI through RAGE increase. Combined injection of sRAGE and hBD-MSCs resulted in enhanced survival of hBD-MSCs and angiogenesis in PIRI-CLI mice. Taken together, AGE-albumin from activated macrophages is critical for both skeletal muscle cell and hBD-MSCs death in PIRI-CLI. Therefore, the inhibition of AGE-albumin from activated macrophages could be a successful therapeutic strategy for treatment of PIRI including CLI with or without stem cell therapy.

## Introduction

Post-ischemic reperfusion injury (PIRI) is associated with the pathogenesis of post-ischemic remodeling in many human and animal organs^1, 2^. Although PIRI occurs in the presence of vascular access, the severity of cell death, organ dysfunction, post-ischemic remodeling and infarct size are similar or worse when compared to the ischemic organs without reperfusion in the cardiovascular, neurologic, and musculoskeletal systems^3–6^. Critical limb ischemia (CLI) is one of the most debilitating sequela of peripheral arterial disease. PIRI has been implicated as one of the underlying pathophysiology of CLI where the skeletal muscle cells in the infarct area are induced to undergo apoptosis and suffer the similar consequence of acute myocardial infarction (AMI) and cerebrovascular accident (CVA)^7, 8^.

Several studies targeted the inflammatory process, however, anti-inflammatory treatment for clinical PIRI did not protect against the host cell death such as cardiomyocytes, skeletal myocytes, or neurons due to the multifactorial complexity of inflammation, involving multiple molecule and cell types^6, 9^. For an example, acute infarction rapidly triggers innate pathways to trigger an inflammatory reaction by secretion of molecules such as high motility group protein 1 (HMGB1) or monocyte chemo-attractant protein 1 (MCP-1)^10–12^. Apoptosis of the majority of host cells follows and the infarct matures with high amounts of fibrosis including collagen fibers^13^. The inflammatory consequences of PIRI include a cascade of diverse cell types and reactions, resulting in newly recruited cells. As the most abundant non-host cell population in the inflammatory site of PIRI, M1/M2 macrophages infiltrate and contribute to the pro-inflammatory milieu in the infarcted area^14–19^. This recruitment of two different populations of monocytes or macrophages in the infarct area has been the subject of many debates on the roles of these cell types. The exact contribution of either cell types remains unclear.

Recently, we have been reported that AGE-albumin (advanced glycation end product), the most abundant AGE product, is synthesized and secreted from activated macrophages and reported as a key inducer of host cell death in various degenerative diseases by increased expression of receptor-AGEs (RAGE)^3, 20–22^. However, there are no reports to show that AGE-albumin is critical in PIRI and the inhibition can protect the host cell death. Recently, stem cell therapy has emerged as a promising method for management of PIRI clinically. However, satisfactory results have not been reported by stem cells in the treatment of PIRI associated with many debilitating human diseases such as AMI, CVA, or CLI due to significant and rapid loss of stem cells in the area of injury^23–26^.

In this study, we hypothesized that AGE-albumin secreted from activated macrophages induces cell death of both the native skeletal muscle cells and the newly introduced stem cells by a RAGE-dependent pathway. Therefore, inhibition of AGE-albumin can protect against the death of skeletal muscle cells and stem cells after PIRI and enhance the recovery of infarcted organs.

## Results

### Post-ischemic reperfusion injury (PIRI) induced macrophage activation and skeletal muscle cell death

**Figure 1:**
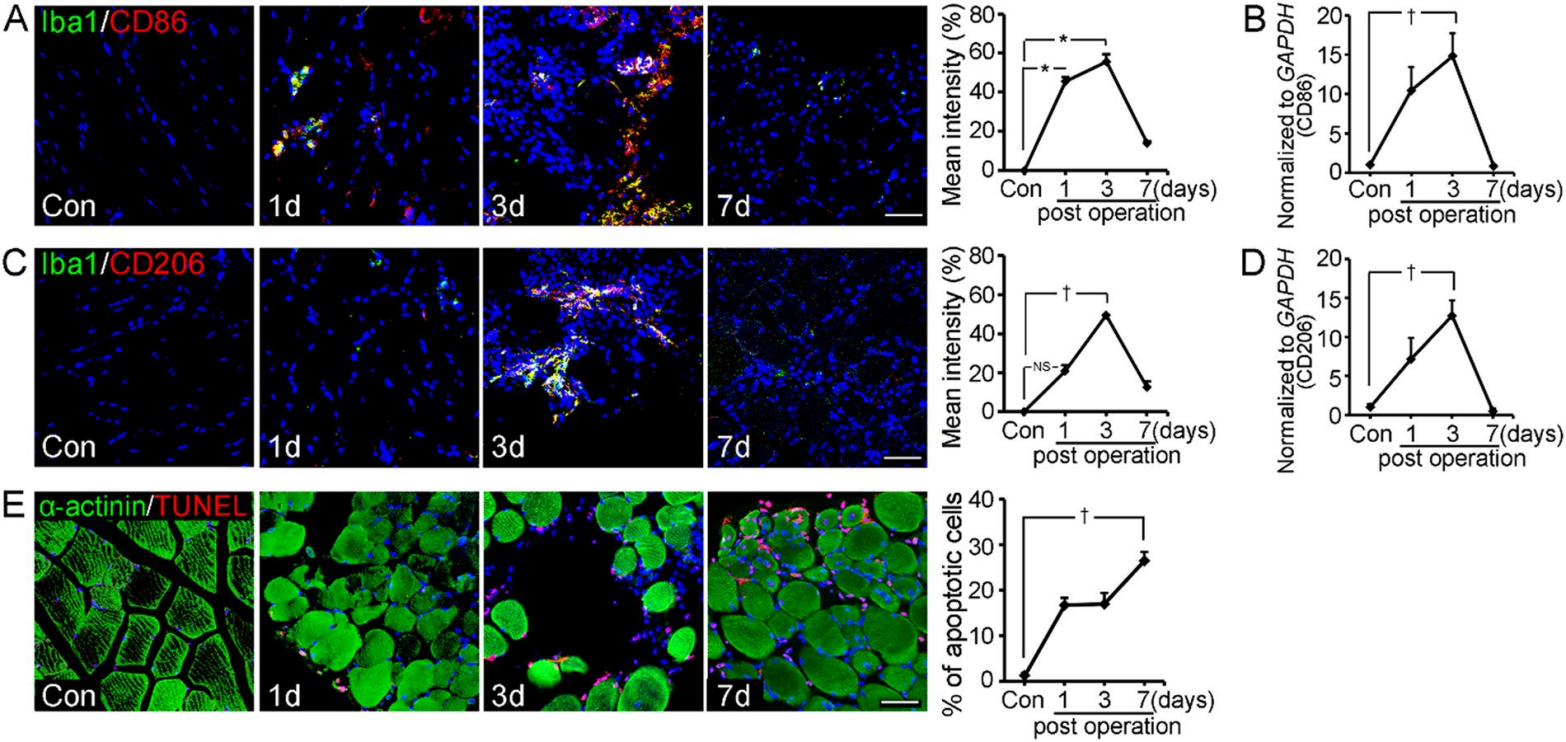
Distribution of activated macrophages and skeletal muscle cell death in post-ischemic reperfusion injury (PIRI) - CLI of mouse. (**A**,**C**) Confocal microscopic images show activated macrophage marker (Iba1, green), M1 macrophage marker (CD86, red), M2 macrophage marker (CD206, red) or nucleus (DAPI, blue). (**B**,**D**) mRNA expression levels of M1 and M2 macrophage were measured by qRT-PCR. (**E**) Apoptotic cells are illustrated by TUNEL (red) with skeletal muscle cell marker (α-actinin, green) and nucleus (DAPI, blue) in a time dependent manner. Scale bar = 50 *μ*m. *mean ± s.d. (P < 0.05) and (^†^P < 0.01) vs. control mice.

We hypothesized that activated macrophages can induce skeletal muscle cell death by advanced glycation end products–albumin (AGE-albumin) and receptor-AGEs (RAGE)^27, 28^. First, we checked the macrophage activation and skeletal muscle cell death in the PIRI-critical limb ischemia (CLI) animal model. Total population of activated macrophages showed a dramatic increase from control (Con) day 1 (1d) to day 3 (3d) and a rapid decrease on day 7 (7d) after PIRI-CLI (Fig. 1A,C). For analysis of the sub-population of activated macrophages, we performed double immunohistochemical staining and qRT-PCR with M1 (CD86)/M2 (CD206)-type specific markers in PIRI-CLI. The number of M1 or M2 macrophages increased from day 1 until day 3 after PIRI-CLI, and then decreased rapidly until day 7 (Fig. 1B,D). However, the number of M1 macrophages was higher than that of M2 macrophages (Fig. 1A–D and Fig. S1A,B). alpha-actinin (α-actinin) immunostaining and TUNEL showed that the level of apoptosis in skeletal muscles was increased from day 1 to 7 in the PIRI-CLI model. The number of apoptotic cells reached to maximum at day 7 and it was around 26.5% of total DAPI-positive cells (Fig. 1E and Fig. S1C). These results confirmed that the number of macrophages increased initially. M1 macrophages were more dominant than M2 macrophages and these cell activations were followed by skeletal muscle cell death in PIRI-CLI models.

### Synthesis and secretion of AGE-albumin from activated M1 macrophages in PIRI-CLI models

**Figure 2:**
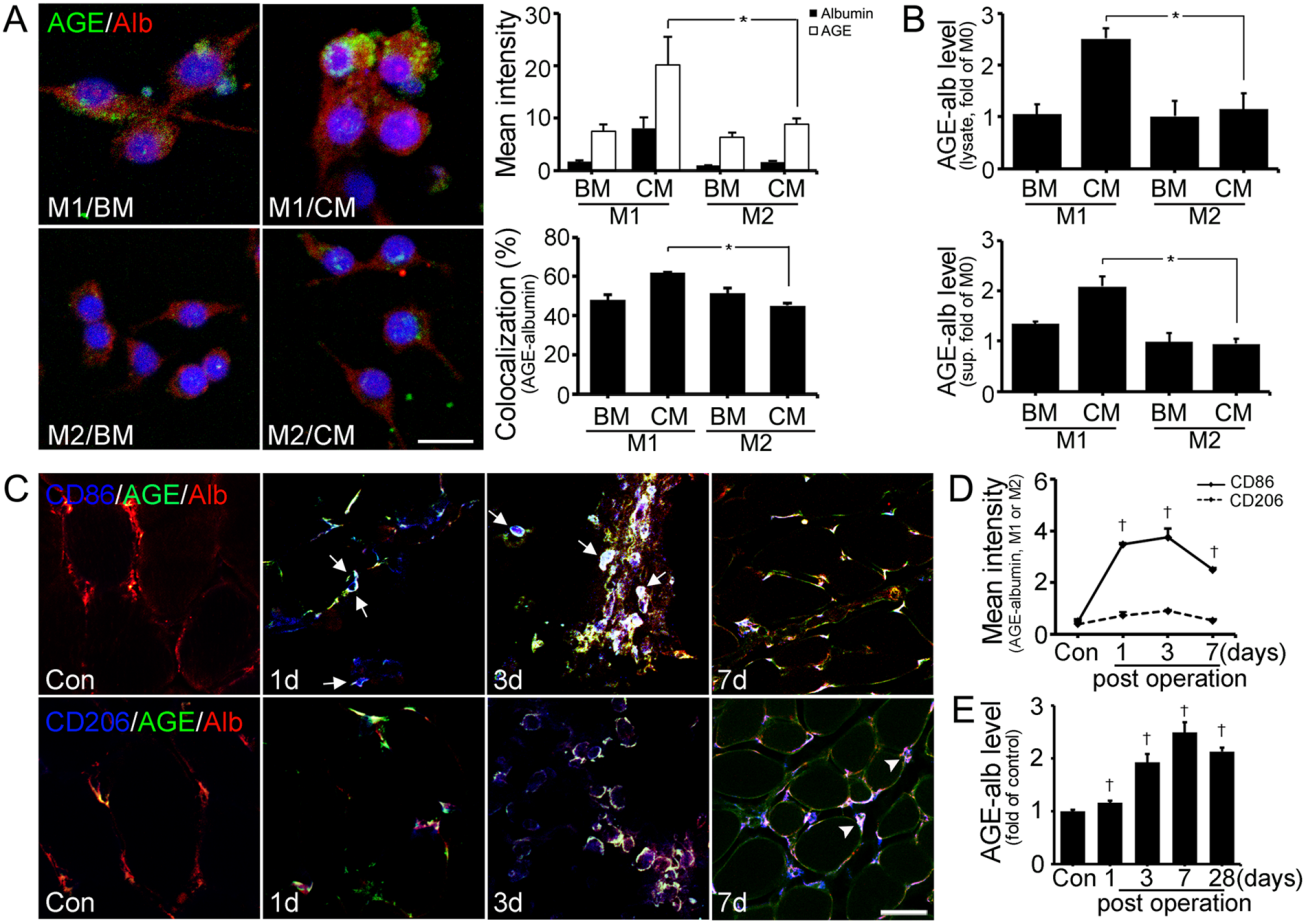
Expression of AGE-albumin in M1/M2 macrophage cells and animal model. (**A**) Triple-labeled confocal microscopic images shows relative levels of AGEs (green), albumin (red), and DAPI (blue) in M1 or M2 cells with basal media (BM) or conditioned media (CM) treatment. Scale bar = 20 *μ*m (**B**) The amounts of intracellular (cell lysate) and extracellular (supernatant) AGE-albumin of macrophage cells were determined by ELISA. (**C**) Triple-labeled confocal microscopic images of AGEs (green) and albumin (red) with activated macrophage markers (blue) were taken. Arrows indicate AGE-albumin positive labeling of macrophages and AGE-albumin is shown in arrowhead. (**D**,**E**) The AGE-albumin positive labeling of macrophages (white) was determined by Zen 2009 software (Zeiss) and the AGE-albumin level of PIRI-CLI damaged skeletal muscle was also detected until 4 weeks. Scale bar = 50 *μ*m. mean ± s.d. (*P < 0.05) and (^†^P < 0.01) vs. Con.

Next, we investigated whether AGE-albumin from activated macrophages is critical in skeletal muscle cell death leading to PIRI-CLI. To determine the expression level of AGE-albumin, triple-labeled fluorescent staining was performed in M1 or M2 macrophages after differentiation from Raw 264.7 cells (Fig. 2A). This labeling demonstrated that AGE-albumin was mostly present in activated M1 macrophages after application of hypoxia and FBS deprivation conditioned medium (CM) to skeletal muscle cells compared to M2 macrophages with or without basic medium (BM) treatment (Fig. 2A and Fig. S2A,B). ELISA data for the cell lysate and medium (supernatant) also showed that AGE-albumin synthesis and its extracellular secretion were significantly elevated in CM treated M1 macrophages compared to CM treated M2 macrophages and these secreted AGE-albumin in M1 macrophages dominantly induced L6 cell death (Fig. 2B and Fig. S3).

For analysis of the distribution of AGE-albumin from activated macrophages in animal models, we performed triple immunohistochemical staining in skeletal muscles after PIRI-CLI in mice (Fig. 2C–E). AGE-albumin was co-localized with activated M1 macrophages (arrow) and its staining intensity was increased from day 1 until day 3 after PIRI-CLI, and then decreased rapidly until day 7. Higher staining intensity was observed in M1 macrophages than in M2 macrophages in PIRI-CLI models similar to the skeletal muscle cells (Fig. 2C,D). Interestingly, the expression level of AGE-albumin was increased from day 1 to 7 compared to control but decreased on day 28 (Fig. 2E). However, the overall level of AGE-albumin increased 2.13 folds than control.

### AGE-albumin induced skeletal muscle cell death via RAGE-MAPK pathway in PIRI-CLI mouse

**Figure 3:**
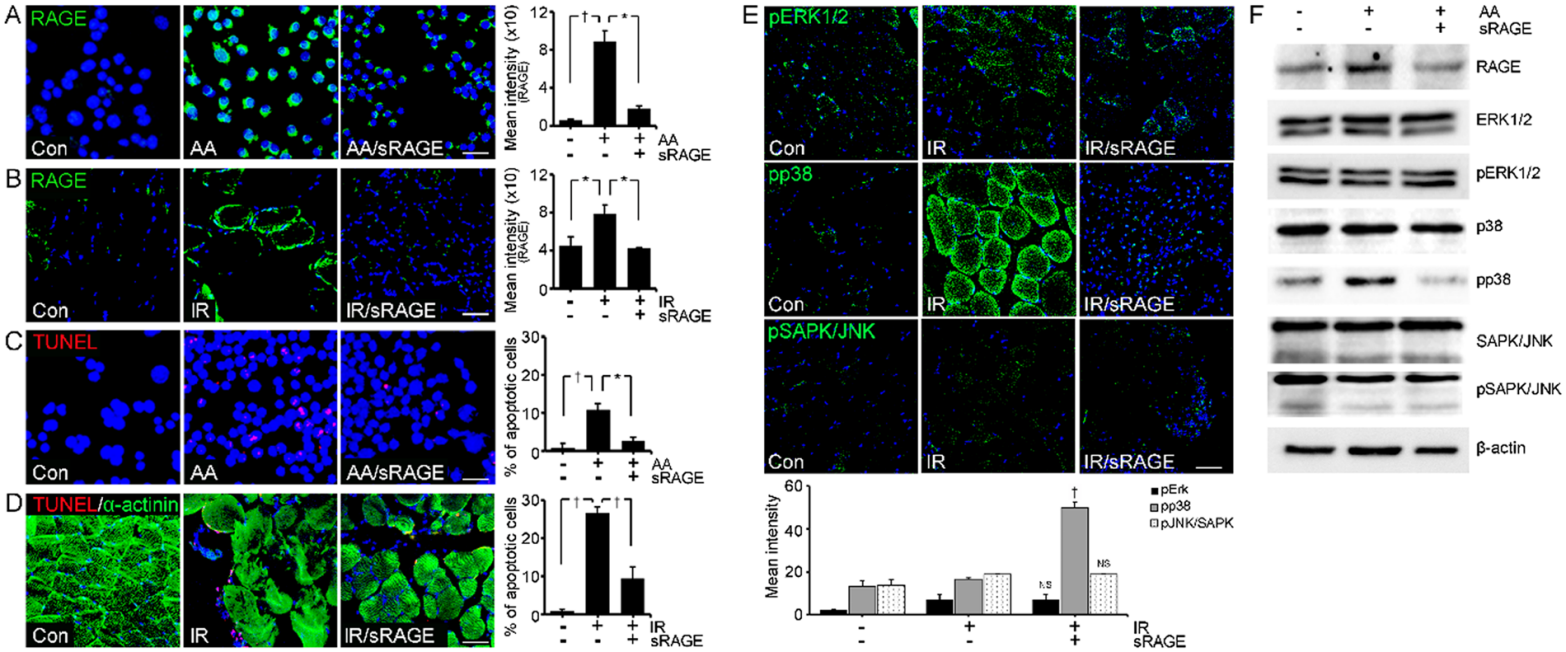
RAGE expression and its inhibition by sRAGE through MAPK pathway. (**A**,**B**) Relative levels of RAGE (green) were determined by immunohistochemistry analysis. The levels of RAGE in L6 cells after AGE-albumin (AA), AA with sRAGE treatment (AA/sRAGE) and in PIRI-CLI (IR) without or with sRAGE treatment. (**C**,**D**) Apoptotic L6 cells (red) and skeletal muscles of PIRI-CLI mice (red) with skeletal muscle cell marker (α-actinin, green) were analyzed by TUNEL labeling. (**E**,**F**) Level of MAPK intensity was determined in mice muscle by immunohistochemistry and Immunoblotting. Scale bar = 50 *μ*m. mean ± s.d. (*P < 0.05) and (^†^P < 0.01).

A receptor protein for AGEs (RAGE) is known to be expressed in skeletal muscles after PIRI, and its increased level is highly correlated with skeletal muscle cell death^29^. Therefore, we assessed the question of whether AGE-albumin from activated macrophages can induce RAGE expression in skeletal muscle and leads to skeletal muscle cell death. Immunohistochemical data showed that the amount of RAGE in skeletal muscle cells was increased after exposure to AGE-albumin (AA) only but decreased significantly after combined treatment of AGE-albumin and soluble RAGE (AA/sRAGE) (Fig. 3A). A similar result was observed in the PIRI-CLI animal model where the amount of RAGE was significantly increased and then significantly decreased when treated with sRAGE, which is soluble form of RAGE (Fig. 3B). TUNEL and immunostaining showed that levels of apoptosis in both skeletal muscle cells and PIRI-CLI animal models also significantly increases and then decreased after treatment with sRAGE (Fig. 3C,D).

Since the stress-activated MAPK is critically important in initiating apoptosis^20, 21^, we checked the changes of the MAPK and RAGE levels in the PIRI-CLI animal model. Immunohistochemical and immunoblot analyses showed that the levels of RAGE and pp38 were significantly increased after PIRI-CLI compared with control, but decreased significantly after treatment with sRAGE in PIRI-CLI (Fig. 3E,F). These results demonstrate that AGE-albumin directly promotes apoptosis of skeletal muscle cells through activation of the RAGE-p38 pathway.

### AGE-albumin inhibition enhanced the survival of hBD-MSCs in the PIRI-CLI mouse

**Figure 4:**
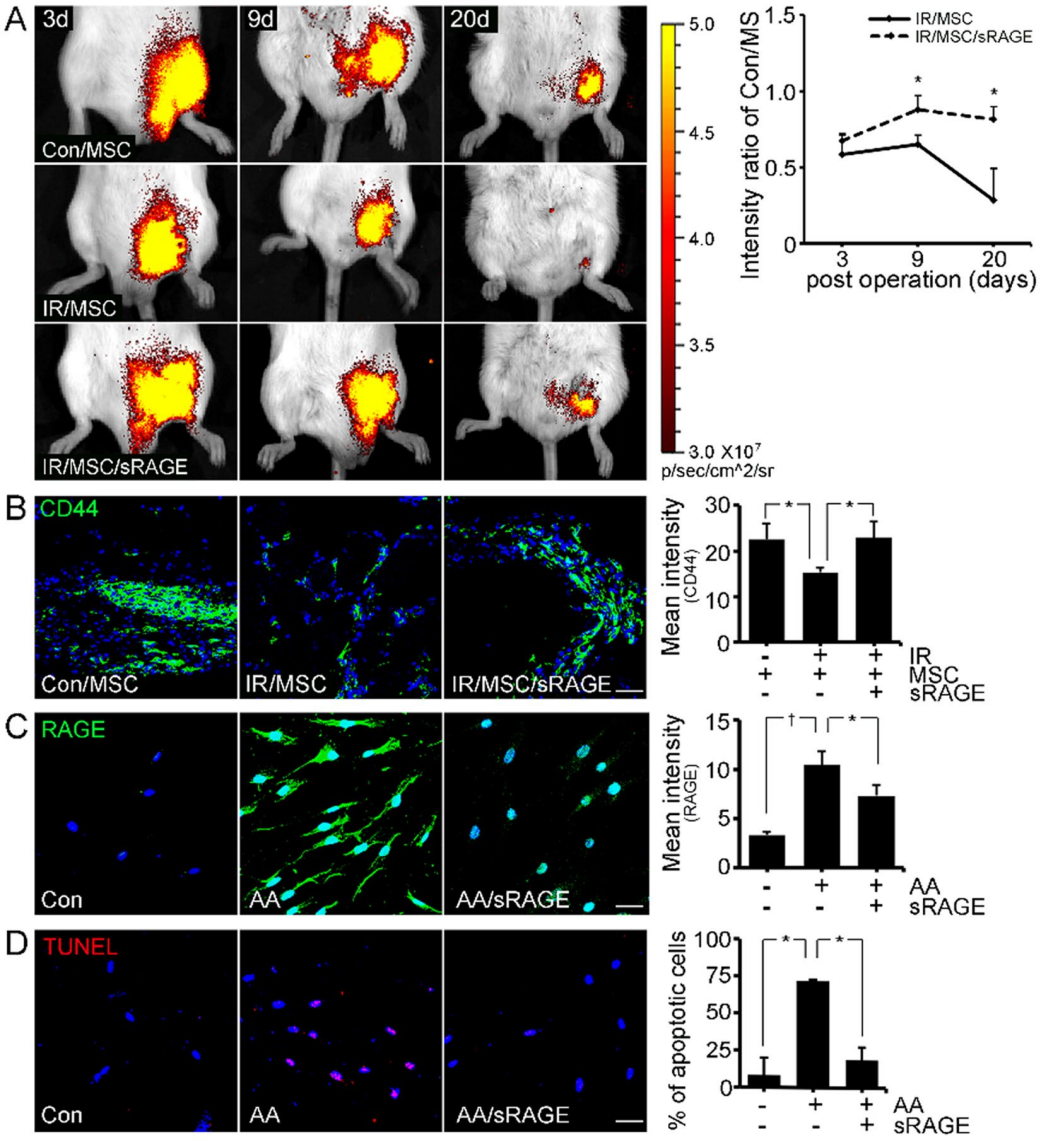
*In vivo* image system and confocal images of survival of human BD-MSC by sRAGE. (**A**) *In vivo* image system (IVIS) images of human BD-MSCs (hBD-MSCs) in PIRI-CLI models with or without sRAGE treatment. The ratio of fluorescent intensity was measured. (**B**) Immunohistochemical staining shows injected human stem cells (CD44) of the cell death. (**C**) Relative levels of RAGE in hBD-MSCs and apoptotic cells (red) were analyzed by Zen 2009 software. Scale bar = 50 *μ*m. mean ± s.d. (*P < 0.05) and (^†^P < 0.01).

To determine the survival rate of hBD-MSCs in the PIRI-CLI mouse, an *in vivo* imaging system (IVIS) was used to visualize the population of living hBD-MSCs. IVIS results showed that the fluorescence intensity ratio was 0.58 and 0.65 on day 3 and day 9, respectively. This decreased rapidly to 0.28 on day 20 in the PIRI-CLI animal model compared to control/hBD-MSCs injected mice. However, the survival rate of hBD-MSCs was dramatically increased to 0.87 and 0.81 on days 9 and 20, respectively, after sRAGE treatment in PIRI-CLI (Fig. 4A).

The survival of injected hBD-MSCs was also evaluated by immunohistochemistry using human MSC specific marker (CD44) in PIRI-CLI mice. The results showed that the level of CD44 positive cells decreased rapidly by 1.48-fold compared to control/hBD-MSCs (Con/MS) injected mice. However, co-treatment with sRAGE in PIRI-CLI animals prevented the decrease significantly by 1.02-fold reduction of hBD-MSCs survival (Fig. 4B).

We also confirmed the effects of AGE-RAGE and hBD-MSCs death *in vitro* after treatment of AGE-albumin with or without sRAGE. The intensities of RAGE and TUNEL increased significantly but decreased dramatically after subsequent co-treatment with sRAGE (Fig. 4C,D). AGE-albumin also induced apoptosis of the injected hBD-MSCs by RAGE increase but AGE-albumin inhibition protected the hBD-MSCs death.

### AGE-albumin inhibition enhanced angiogenesis after hBD-MSC treatment in PIRI-CLI animals

**Figure 5:**
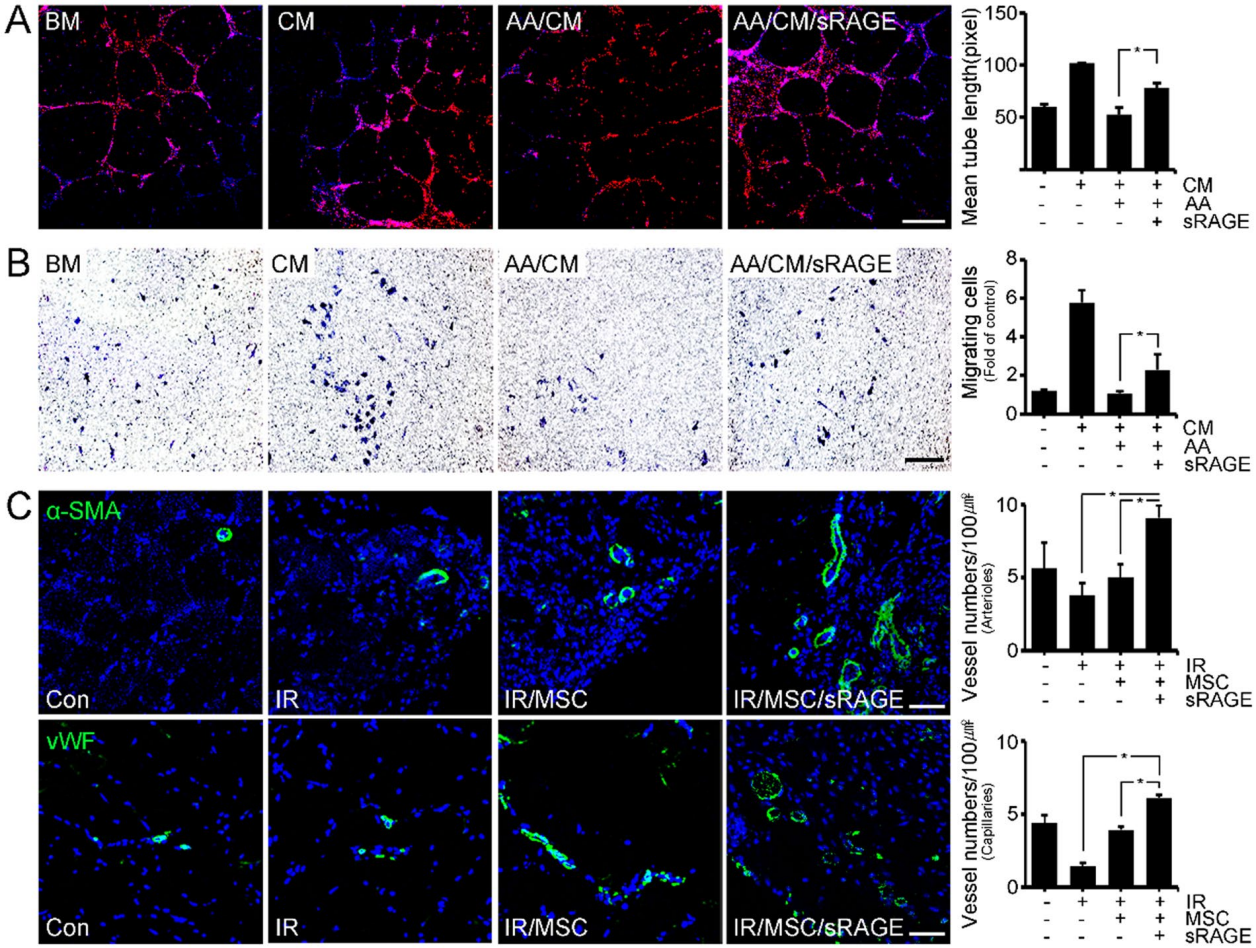
Induction of angiogenesis by hBD-MSCs and sRAGE in PIRI-CLI. (**A**,**B**) HUVECs were treated with various conditioned medium with BM, CM, AGE-albumin with CM (AA/CM), and sRAGE with AA/CM (AA/CM/sRAGE). (**A**) Formed tube of HUVECs stained with endothelial cell markers (Ac-LDL, red) and nuclei (DAPI) and the tube length was measured using the Wimasis image analysis website. scale bar = 50 *μ*m (**B**) HUVECs migrated to the opposite side of the culture insert and the migrating cells were stained with hematoxylin and the number of migrated cells was counted using Image J (NIH) software. scale bar = 25 *μ*m (**C**) To validate angiogenesis *in vivo*, alpha smooth muscle actin (α-SMA, green) and von Willebrand factor (vWF, green) were stained with blood vessels of PIRI area. The numbers of α-SMA and vWF were counted using Zen 2009 software. Scale bar = 50 *μ*m. mean ± s.d. (*P < 0.05).

To determine whether angiogenesis is induced by hBD-MSCs or its secretomes, we investigated the degree of tube formation and migration using HUVECs after hBD-MSCs treatment. Both the tube formation and migration of HUVECs were increased after treatment of cultured medium of hBD-MSC (Fig. 5A,B). However, the degree was decreased after AGE-albumin treatment, but increased dramatically after co-treatment with sRAGE (Fig. 5A,B). Next we checked the expression of angiogenesis markers such as alpha smooth muscle actin (α-SMA) for arterioles and von Willebrand factor (vWF) for visualizing capillary formation (Fig. 5C). Confocal microscopy showed that the population of both arterioles (α-SMA) and capillaries (vWF) was increased by 2.78 and 1.33 folds after hBD-MSC injection in PIRI areas of leg muscle of mice. However, these 2 cell populations were further enhanced dramatically again by 4.33- and 2.42 folds after co-treatment with hBD-MSCs and sRAGE to inhibit AGE-albumin in the leg muscles of PIRI-CLI animals (Fig. 5C).

### AGE-albumin inhibition protected against skeletal muscle death after hBD-MSC treatment in PIRI-CLI mice on 4 weeks

**Figure 6:**
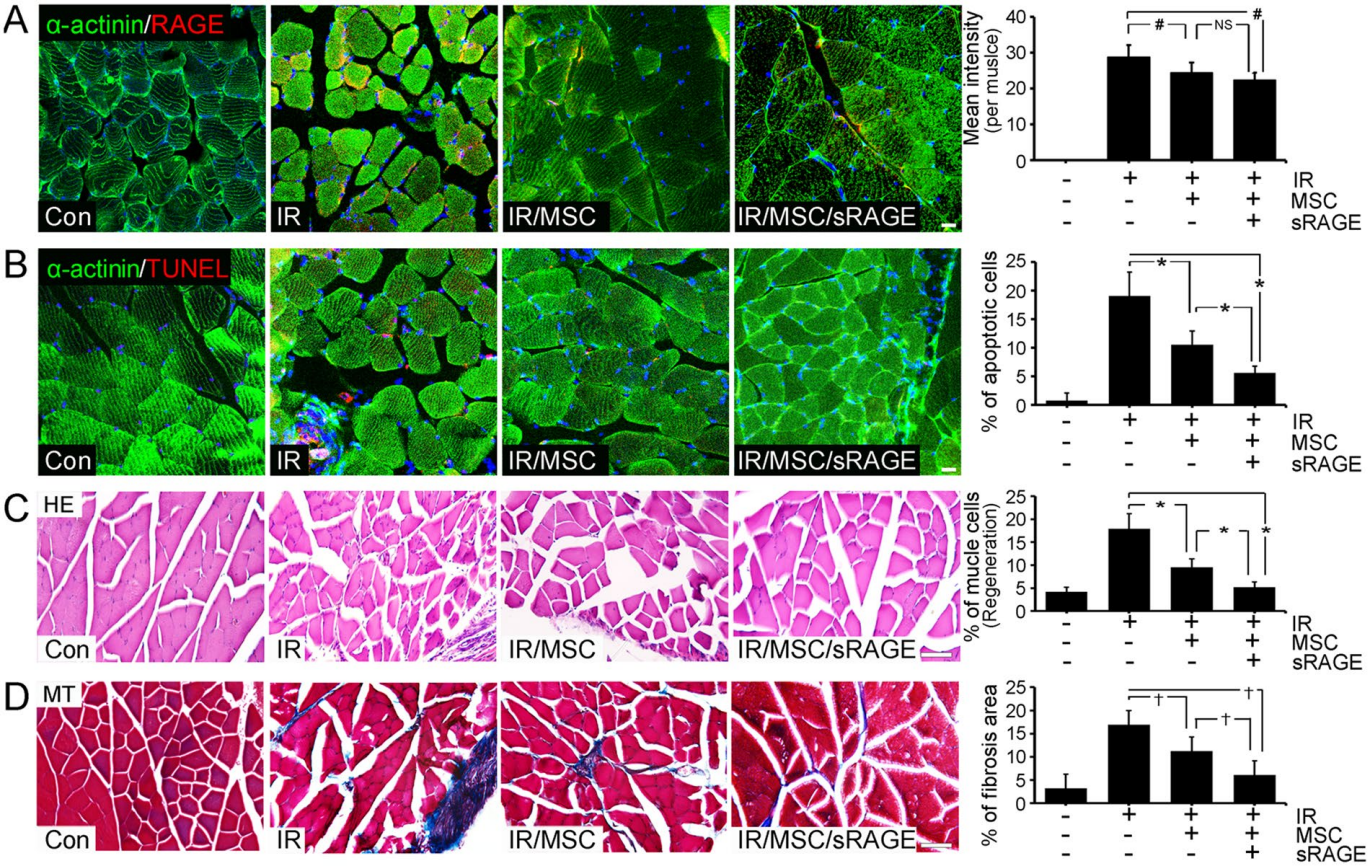
Reduced RAGE expression by sRAGE treatment in PIRI-CLI on 4 weeks. (**A**) The relative levels of RAGE (red) in skeletal muscle cells (α-actinin, green), after hBD-MSCs with (IR/MSC/sRAGE) or without sRAGE treatment (IR/MSC) in PIRI-CLI mouse model. The damaged muscles were evaluated by triple confocal microscopic analyses. (**B**) Apoptotic cells were illustrated by TUNEL (red) in skeletal muscle cells (green), after hBD-MSC treatment with or without sRAGE treatment. Scale bar = 20 *μ*m (**C**,**D**) The damaged skeletal muscles were stained by hematoxylin and eosin staining (HE) and fibrotic area was evaluated by Masson’s trichrome (MT) staining in PIRI-CLI mice on 4 weeks. Scale bar = 50 *μ*m. mean ± s.d. (*P < 0.05) (^†^P < 0.01) and (^#^P < 0.001).

AGE-albumin inhibition protected against skeletal muscle death after hBD-MSC treatment in PIRI-CLI mice on 4 weeks. We performed immunohistochemical analyses to determine whether sRAGE can enhance the survival of skeletal muscle cells for hBD-MSCs treatment in PIRI-CLI mice on 7 days (Fig. S4) and 4 weeks. Immunohistochemical data showed that the labeling intensity of RAGE was increased in PIRI-CLI and remained similar after hBD-MSCs treatment. However, the labeling intensity decreased dramatically after co-treatment of hBD-MSCs with sRAGE in PIRI-CLI animals (Fig. 6A). The labeling intensity of TUNEL was also increased in PIRI-CLI but decreased after hBD-MSCs treatment. However, further significant decrease was observed after co-treatment of hBD-MSCs with sRAGE in PIRI-CLI animals (Fig. 6B).

To investigate the protective effect in tissue level of hBD-MSC and AGE-albumin inhibition in skeletal muscle cell death, we evaluated the morphology of the skeletal muscles in PIRI-CLI mice. Hematoxylin and eosin (H&E) staining showed that the number of the damaged muscle cells, demonstrated by nuclear atypia, was decreased by 1.88-fold after hBD-MSC treatment, which was further dramatically decreased by 3.46-fold by co-treatment of hBD-MSC with sRAGE compared to the PIRI-CLI animals (Fig. 6C). Masson’s trichrome staining also showed the decrease in fibrotic tissue after hBD-MSC treatment compared to control but decreased dramatically after co-treatment with sRAGE in PIRI-CLI animals (Fig. 6D) and in low dosage of hBD-MSC treatment, sRAGE more improves the protective effect of hBD-MSC compared to without sRAGE (Fig. S5).

Together, our data showed that AGE-albumin from activated M1 macrophages induces skeletal muscle cell death and the inhibition of AGE-albumin promoted hBD-MSC survival and protected the severity of PIRI-CLI.

## Discussion

Many investigators have reported the recruitment of activated macrophage cells in various post-ischemic reperfusion injury areas^16, 30^. However, it is poorly understood whether activated macrophages promote skeletal muscle cell damage or not. We recently reported that AGE-albumin can be synthesized in activated mononuclear phagocytic cells in several areas of the body including brain and lung^31, 32^. We also demonstrated that the synthesis and extracellular secretion of AGE-albumin from microglial cells were elevated upon activation and is critical for host cell death^20, 33^.

Similarly, we first hypothesized that activated macrophages can induce skeletal muscle cell death based on an increase in AGE-albumin and RAGE in PIRI-CLI. First we checked the macrophage activation and skeletal muscle cell death in the PIRI-CLI animal model. Our result of alpha actinin and TUNEL assay showed that human skeletal muscle cell death increased gradually in the PIRI-CLI model. The numbers of M1 macrophages increased more than those of M2 macrophages on day 3 after PIRI-CLI. Then, the numbers of both macrophage cell types showed a rapid decrease until day 7 after PIRI-CLI. According to these results the total number of macrophages increased initially, the M1 macrophages were more dominant than M2 sub-population, and the end-result was skeletal muscle cell death. These findings are similar to several other observations of macrophage recruitment, activation and host cell death in PIRI such as acute myocardial infarction and stroke^18, 34, 35^. In addition, triple fluorescent labeling and immunoblot analyses showed that the AGE-albumin synthesis in M1 macrophage cells^36^ and its extracellular secretion were significantly elevated compared to M2 macrophages in PIRI-CLI animals. Based on these findings, we could conclude that the synthesis of AGE-albumin in M1 macrophage cells and its extracellular secretion are closely related to increased skeletal muscle death in PIRI-CLI. This result is consistent with those of several reports on the more deleterious role of M1 macrophages in the inflammatory site than M2 macrophages^36^.

A receptor protein for AGEs (RAGE) is known to be expressed in the muscles after PIRI and its increased level is highly correlated with muscle cell death^10, 37–39^. Therefore, we also assessed the question of whether AGE-albumin from activated macrophages increases the expression of RAGE, leading to skeletal muscle cell death. Our data demonstrated that the amounts of RAGE in human skeletal muscle cells were increased upon exposure to AGE-albumin but showed a rapid decrease after treatment with sRAGE, an AGE inhibitor. The PIRI-CLI animal result showed that the RAGE increase induced skeletal muscle cell death while sRAGE inhibited such death. This AGE-RAGE dependent cell death has been well established by many reports including AMI, stroke, and other degenerative diseases. Remodeling after PIRI undergoes the same RAGE dependent muscle cell death^40–43^. In terms of the mechanism of cell death after RAGE increase, immunoblot analysis showed that the level of pp38 was significantly increased in both cell and animal models exposed to AGE-albumin compared to control. But the level of pp38 was decreased after exposure of human skeletal muscle cells to AGE-albumin and sRAGE. These data demonstrate that AGE-albumin directly promotes apoptosis of neuronal cells through activation of the RAGE-p38 pathway. This is also similar to microglial activation in degenerative diseases in brain and explains further how mononuclear phagocytic cells are activated^21^.

Second, we hypothesized that stem cell survival is also limited due to AGE-albumin from activated macrophages and hardly improve the recovery of PIRI. We used an in vivo imaging system (IVIS) to determine the survival rate of hBD-MSC in the PIRI-CLI mouse model. The population of viable hBD-MSCs decreased rapidly in the PIRI-CLI animal model but increased dramatically after AGE-albumin inhibition. The result was confirmed by immunohistochemistry using human MSC specific marker (CD44) in both hBD-MSCs and skeletal muscles of PIRI-CLI mice. Immunohistochemical data also showed that RAGE labeling was increased in the hBD-MSCs in animal models of PIRI-CLI. The labeling intensity showed a dramatic decrease after co-treatment of hBD-MSCs with sRAGE. These results strongly indicate that the main cause of death of injected hBD-MSCs and skeletal muscle cells is due to increase in RAGE and AGE-albumin from activated M1 macrophages in PIRI. There are many reports supporting this finding that the inhibition of AGE-albumin or RAGE can protect against tissue damage^44, 45^. Our result showed that the origin of AGE-albumin is activated macrophages and its inhibition can protect against tissue damage as well as hBD-MSC survival in PIRI-CLI.

To determine the angiogenic capacity of hBD-MSCs and their improved survival by AGE-albumin inhibition, we first measured the size of blood vessels in PIRI-CLI animals. The size increased after BD-MSC injection in leg muscles and was further enhanced dramatically after co-treatment with sRAGE for AGE-albumin inhibition in the leg muscles of PIRI-CLI animals. This was confirmed by immunofluorescent confocal microscopy of vWF- and α-SMA-staining in leg muscles of PIRI-CLI mice. The result of the microscopy was consistent, which demonstrated increased immunostain of both markers and subsequent dramatic increase after treatment with hBD-MSC and sRAGE in leg muscles of PIRI-CLI animals. AGE-albumin is critical in determining the survival of hBD-MSC. Its *in vivo* inhibition dramatically enhanced the survival of skeletal muscles and angiogenic capacity in PIRI-CLI animals.

Finally, we checked the recovery of skeletal muscles by the AGE-albumin inhibition of hBD-MSCs in the PIRI-CLI model. The number of muscle fibers increased after hBD-MSC treatment but it further enhanced dramatically after co-treatment with sRAGE. Masson’s trichrome staining also showed a decrease in interstitial tissue after hBD-MSC treatment compared to control but dramatically decreased further after co-treatment with sRAGE. TUNEL staining also showed that the number of apoptotic muscle cells was decreased after hBD-MSC treatment but similarly demonstrated a dramatic decrease after treatment with sRAGE in leg muscles of PIRI-CLI animals.

In summary, our data show for the first time that AGE-albumin is synthesized mainly in activated M1 macrophage cells and secreted into areas of PIRI-CLI mice to induce the death of both skeletal muscle cells and newly injected hBD-MSCs. The inhibition of AGE-albumin promoted the survival of both skeletal muscle cells and hBD-MSCs in PIRI-CLI and enhanced angiogenic activity to protect against skeletal muscle injury in PIRI. As well as local effects, sRAGE-MSC reduced systemic reaction such as kidney or liver malfunction (Fig. S6).

Our results, therefore, provide a new mechanistic insight by which activated macrophage cells play an important role in promoting muscle cell death in PIRI-CLI by synthesizing and secreting potentially toxic AGE-albumin. Co-treatment of hBD-MSCs with AGE-albumin inhibition could be an excellent therapeutic strategy for many human diseases of PIRI.

## Methods

### Animals

Seven weeks of male Balb/c mice were purchased from Orient Bio (Seongnam, Korea) and these mice were maintained on a light/dark cycle (12 hrs/12 hrs) with adequate access to food and water for at least one week prior to their usage. All animal experiments were approved by the Institute Animal Care and Use Committee (AAALAC International; approval number: LCDI-2013-0056) of Lee Gil Ya Cancer and Diabetes Institute of Gachon University.

### Generation of PIRI-CLI animal

The mice were anesthetized with a mixture of 2% xylazine hydrochloride (Rompun, 5–10 mg/kg; Bayer) and tiletamine/zolazepam (Zoletil 50, 25 mg/kg, Virbac Animal Health) through intra-peritoneal injection. After induction of anesthesia, surgical procedures were performed by following protocol. Briefly, longitudinal incision of left thigh skin was made. Proximal and distal ends of the femoral artery was ligated. Complete femoral artery occlusion was achieved and continued for 1 hr. The ligation was removed and blood reperfusion was allowed into the ischemic muscle. After recovery of blood supply to the damaged muscles, the following 3 treatment arms were established: 1) 8 μl normal saline, 2) 1 × 10^6^ human bone marrow-derived mesenchymal stem cells (hBD-MSCs), and 3) hBD-MSCs with 0.8 μg/8 μl soluble RAGE recombinant protein treatment (sRAGE; Biovendor, Brno, Czech Republic). These agents were injected directly into the PIRI-CLI muscles.

### In vivo imaging system (IVIS)

One million hBD-MSCs were stained with Vivo track 680 fluorescence reagent (Perkin Elmer, Norwalk, CT) according to the manufacturer’s instructions. Briefly, hBD-MSCs were incubated with Vivo track 680 reagent for 15 mins at room temperature in the dark. After washing with 0.1 M phosphate buffered saline (PBS; Gibco, Grand Island, NY), the labeled hBD-MSCs with or without sRAGE were injected directly into the damaged muscle. Grafted hBD-MSCs were checked by fluorescence intensities from 1 to 20 days after injection. The fluorescence intensity was detected by In Vivo Imaging System (IVIS spectrum, Perkin Elmer) and the expression level was quantified.

### Cell culture and treatment

hBD-MSCs were passaged 5–6 times and cultured in Dulbecco’s modified Eagle’s medium (DMEM, Gibco) containing a low glucose concentration supplemented with 10% fetal bovine serum (FBS, Gibco) and 1% gentamycin. Then, hBD-MSCs were incubated with basal medium (BM, Gibco) with serum (conditioned medium; CM) and with 800 *μ*g/${\rm{m}}\ell$ AGE-albumin (AA/CM) with or without 400 ng/${\rm{m}}\ell$ sRAGE (AA/CM/sRAGE) for two days. These medium were collected in a conical centrifuge tube and cell debris was discarded. Supernatants were concentrated by Amicon centrifugal filter devices (Millipore, USA) following the manufacture’s protocol.

Human umbilical vein endothelial cells (HUVECs; CEFObio, Seoul, Korea) were cultivated with EGM^TM^-2 growth medium including bovine brain extract with heparin, human epithelial growth factor, hydrocortisone and GA-1000 (Lonza, Walkersville, MD) in a 1% gelatin (Sigma-Aldrich, St Louis, MO) coated culture dish. Murine macrophage cells (Raw 264.7) and rattus norvegicus skeletal muscle cells (L6) were also used in this study. These cells were purchased from Korean Cell Line Bank and cultured in DMEM with a high glucose concentration supplemented with 10% FBS and 100 U/${\rm{m}}\ell$ penicillin and 100 μg ${\rm{m}}\ell$ streptomycin. All cells were maintained in a 5% CO_2_ humidified incubator at 37 °C.

### Hypoxia and glucose deprivation in cells

To mimic ischemia reperfusion condition of animal, L6 cells were treated with serum free DMEM medium and were incubated with in a hypoxia chamber filled with a gas mixture consisting 1% O_2_, 5% CO_2_, and 94% N_2_ for 24 hrs. Finally, the treated cells and supernatant were collected.

### Immunohistochemistry

Muscle tissues were fixed with 4% paraformaldehyde in PBS, and then cryoprotected in 30% sucrose at 4 °C overnight. The tissue sections (10 *μ*m) were performed on a cryostat (Leica CM 1900; Leica, Milton Keynes, UK). Ten percent normal goat serum was used for blocking of non-specific binding at 4 °C overnight. The tissue sections were incubated with the primary antibodies (see Supplementary Table S1) and then rinsed with PBS and they were incubated with fluorescence conjugated secondary antibodies. All secondary antibodies are also listed in Table S1; these antibodies were incubated for 1 hr in the dark. Nuclei were counterstained by 4′6-diamino-2-phenilindole (DAPI, Sigma-Aldrich) at room temperature for 30 secs. After washing PBS, sections were mounted and coverslipped on glass slides using Vectashield mounting medium (Vector Laboratories, Burlingame, CA) and analyzed by confocal microscopy (Carl Zeiss 710, Carl Zeiss, Germany).

### Immunocytochemistry

Cells were grown on 8-well Lab-Tek II chamber slides (Life Technologies, Gaithersburg, MD), washed with PBS, fixed with methanol for 15 mins on ice, and washed again with PBS. After fixation, these cells were incubated with primary antibodies at 4 °C overnight. The cells were rinsed with PBS, followed by incubation with secondary antibodies at room temperature for 1 hr in the dark. The antibodies used for this study are listed in Table S1. After washing secondary antibodies with PBS, coverslips were mounted by using the Vectashield mounting medium and the fluorescence was examined under a confocal microscope (Carl Zeiss 710).

### Immunoblotting

To validate relationship between AGE-albumin and mitogen-activated protein kinases (MAPK) in skeletal muscle, L6 cells were firstly exposed to 400 ng/${\rm{m}}\ell$ sRAGE for 36 hrs with or without 800 *μ*g/${\rm{m}}\ell$ AGE-albumin for 48 hrs. Effects of sRAGE treatment to L6 cells in hypoxia and glucose deprivation were confirmed by immunoblotting. L6 lysates were isolated by RIPA lysis buffer (TaKaRa, Tokyo, Japan). Concentration of protein samples was measured using the Bicinchoninic acid (Thermo Scientific, Rockford, IL). Equal amounts of proteins (30 *μ*g protein/lane) were separated by 8–12% sodium dodecyl sulfate polyacrylamide gel electrophoresis (SDS-PAGE) and then transferred to polyvinylidene fluoride (PVDF). PVDF membrane was incubated with appropriate diluted primary antibodies at 4 °C overnight. The membrane was washed with tris buffered saline with 1% tween 20 (TTBS) three times and incubated with secondary antibodies for 1 hr at room temperature. The primary and secondary antibodies are listed in Table S1. The blotting membrane was developed with enhanced chemiluminescence (ECL) on LAS-4000 (Fuji Film, Tokyo, Japan).

### Quantitative polymerase chain reaction (qRT-PCR)

RNA was isolated from each group by using Trizol reagent (Invitrogen, Carlsbad, CA), and cDNA was synthesized by using a PrimeScript 1st strand cDNA Synthesis Kit (TAKARA) according to the manufacturer’s protocol. qRT-PCR was performed by using CFX384 Touch™ Real-Time PCR detection system (Bio-Rad, Hercules, CA) and, reaction efficiency and threshold cycle number were determined by using CFX Manager™ Software. All reacted primers are shown in Table S2.

### Cell viability assay

WST (Water-soluble tetrazolium salts) was purchased from Daeil Lab Service (Seoul, Korea). To analyze skeletal muscle cell viability using AGE-albumin^27, 28^, 5,000 cells of L6 per well were seeded in 96 well culture plate (SPL Life Sciences, Korea) and conditioned medium from M1 or M2 macrophages was directly treated to L6 cell for 48 hrs in a 5% CO_2_ humidified incubator at 37 °C. WST was mixed with serum free DMEM with high glucose (1:9, v/v, 200 ${\rm{m}}\ell$/well) and then mixture was incubated for 4 hrs in humidified incubator. Optical density was measured by plate reader (Molecular Devices) at 450 nm.

### Sandwich enzyme-Linked Immunosorbent Assay (ELISA)

For measurement of secreted and synthesized AGE-albumin from the activated macrophages and PIRI-CLI injured skeletal muscles, a 96 well-plate was coated with 1 *μ*g/${\rm{m}}\ell$ of albumin antibody in coating solution including 100 mM carbonate/bicarbonate buffer (pH 9.6) at 4 °C overnight. After rinsing with PBS, remaining protein-binding sites were blocked with 5% skim milk for 1 hr at room temperature. Cell lysates and supernatants were added to each well and incubated at 37 °C for 90 mins and at 4 °C overnight. After thoroughly washing the wells, 1 *μ*g/${\rm{m}}\ell$ of AGE antibody was added to each well for 2.5 hrs at room temperature. HRP conjugated anti-rabbit secondary antibody was added to each well at room temperature for 1 hr and the samples in each well were developed with 3,3′,5,5′-Tetramethylbenzidine (TMB) solution (Sigma-Aldrich) for 15 mins, and mixed with the same volume of stop solution (2 M H_2_SO_4_) and the optical density was read at 450 nm using an ELISA plate reader (Molecular Devices, Sunnyvale, CA).

### Angiogenesis assay

HUVECs were stained with acetylated low-density lipoprotein (Invitrogen) as endothelial cell specific marker and conjugated with DiI fluorescent dye according to the manufacturer’s protocol. To remove FBS effects, HUVECs were incubated with medium containing 1% FBS without growth factors for 24 hrs. Tube formation capacity was estimated in matrigel tube formation assay. Growth factor-reduced matrigel (BD Biosciences, San Diego, CA) was spread onto a 24-well plate (Invitrogen) and incubated for 40 mins at 37 °C. HUVECs (5.0 × 10^4^ cells/well) were seeded on solidified matrigel and were treated with BM, CM, AA/CM, or AA/CM/sRAGE, respectively. After 20 hrs of incubation, the images were captured by fluorescent confocal microscopy (Carl Zeiss 710).

To confirm chemotactic motility of hBD-MSCs, transwell migration of HUVECs was implemented. HUVECs (1.4 × 10^5^ cells/well) were seeded on a 24-well transwell plate and treated with BM, CM, AA/CM, or AA/CM/sRAGE for 48 hrs. Un-migrated HUVECs were discarded and hematoxylin stained migrated HUVECs were counted on light microscopy (CX21; Olympus, Tokyo, Japan).

### Histological validation

To confirm change of muscle regeneration, tissues were stained with hematoxylin and eosin. 10 µm sectioned tissues were immerged with 100% zylene, followed by hydration steps using serial ethanol solution. The hydrated muscle tissues were incubated with Mayer’s hematoxylin (Sigma-aldrich) for 1 min and then, washed with running tap water for 3 mins. The tissues were immerged within Eosin Y solution (Sigma-Aldrich) for 20 secs and were finished with serial ethanol step and zylene.

To determine fibrosis area in the PIRI-CLI injured muscles, we performed Masson’s Trichrome staining (Sigma-Aldrich) and all components was used in commercial kit. Briefly, put skeletal muscle slides in the bouin’s solution for 15 mins at 56 °C for fixation and the slides were stained with Iron hematoxylin for 5 mins to nucleus. Biebrich Scarlet was used to stain acidophilic components and Phosphomolybdic-phosphotungstic acid was used for differentiating tissues. Finally, collagen of fibrosis area was stained by Aniline blue.

All stained slides were immersed in gradient alcohol and 100% of xylene and the slides were sealed with cover glass by using DPX medium (Sigma-Aldrich) and these were analyzed on light microscope.

### Statistical analysis

Given the small samples, non-parametric analysis was used in this study. SPSS version 22 (IBM Corporation, Armonk, NY) statistical program was used and Mann-Whitney U test was used to compare groups. P values < 0.05 (*), <0.01 (†), <0.001 (#) were deemed significant. All results are expressed as the mean ± standard deviations (s.d.).

## Electronic supplementary material

Supplementary figures and tables
