# Supplementary material for: Advanced glycation end-product (AGE)-albumin from activated macrophage is critical in human mesenchymal stem cells survival and post-ischemic reperfusion injury
Source: Sci Rep. 2017 Sep 14;7:11593. doi: 10.1038/s41598-017-11773-1 (PMC5599509; doi:10.1038/s41598-017-11773-1)
Supplement: Supplementary file 1 — Supplementary figures and tables [file 41598_2017_11773_MOESM1_ESM.pdf]

Advanced glycation end-product (AGE)-albumin from activated macrophage is critical in human mesenchymal stem cells survival and post-ischemic reperfusion injury

Myeongjoo Son[1,2,¶], Woong Chol Kang[3,¶], Seyeon Oh[2], Delger Bayarsaikhan[2], Hyosang Ahn[1,2], Jaesuk Lee[2], Hyunjin Park[1,2], Sojung lee[1,2], Junwon Choi[1,2], Hye Sun Lee[2], Phillip C. Yang[4], Kyunghee Byun[1,2,*], Bonghee Lee[1,2,*]

[1] Department of Anatomy & Cell Biology, Graduate School of Medicine, Gachon University, Incheon 21936, Republic of Korea

[2] Center for Genomics and Proteomics & Stem Cell Core Facility, Lee Gil Ya Cancer and Diabetes Institute, Gachon University, Incheon 21999, Republic of Korea.

[3] Cardiology, Gachon University Gil Medical Center, Incheon 21565, Republic of Korea

[4] Department of Cardiovascular Medicine, Stanford University, Stanford, CA, 94305, USA

Supplementary figures

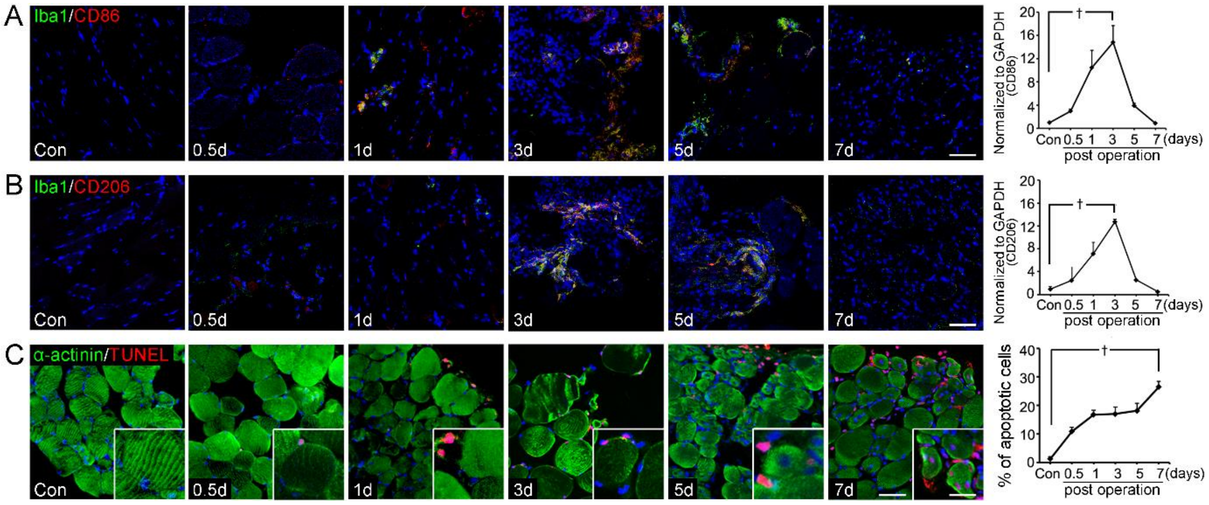

Figure S1. Confirmation of relationship between M1/M2 macrophage activation time and skeletal muscle cell death

(A) Confocal microscopic images illustrate activated M1 macrophage marker (Iba1/CD86, yellow) and activated M2 macrophage marker (Iba1/CD206, yellow) or nucleus (DAPI, blue) until 7 days after reperfusion. (B) mRNA expression levels of M1 (CD86), and M2 (CD206) macrophage were confirmed by qRT-PCR. (C) Apoptotic skeletal muscle cells were illustrated by TUNEL (red) in a time dependent manner. Scale bar = 50 ㎛. mean ± s.d. †, (P<0.01) vs. control mice.

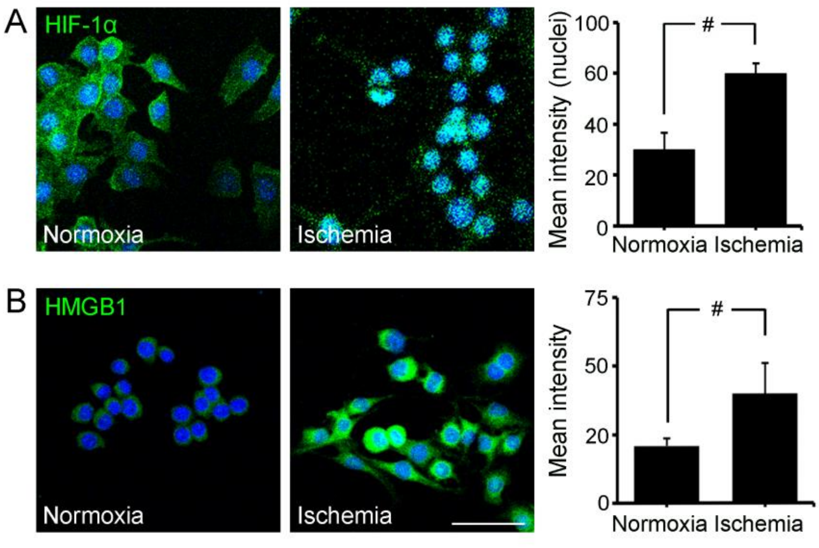

Figure S2. Validation of hypoxia and serum deprivation treated L6 cells

(A, B) Double-labeled confocal microscopic images show fluorescence levels of HIF-1α (green, top), HMGB1 (green, bottom) and DAPI (blue) in L6 cells. Graph is processed from fluorescence intensity and it indicates differences level of hypoxia related proteins after hypoxia and serum deprivation treatment (ischemia) Scale bar = 50 ㎛. mean ± s.d. #, ($P < 0.001$)

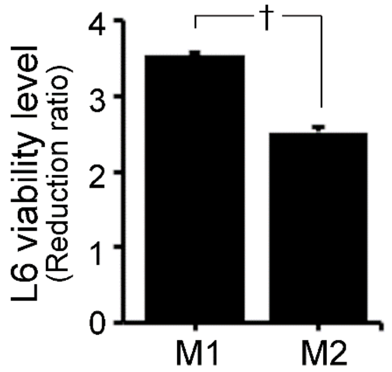

Figure S3. Skeletal muscle cell viability after supernatant treatment from M1 or M2 macrophage

This graph shows M1 or M2 extracellular secretion after conditioned medium (C.M.) treatment for 48 hrs decreased L6 cell number as determined by cell viability assay. Especially, the number of L6 cells after M1 C.M. treatment was significantly decreased compared to M2 C.M treatment. mean ± s.d. †, ($P<0.01$)

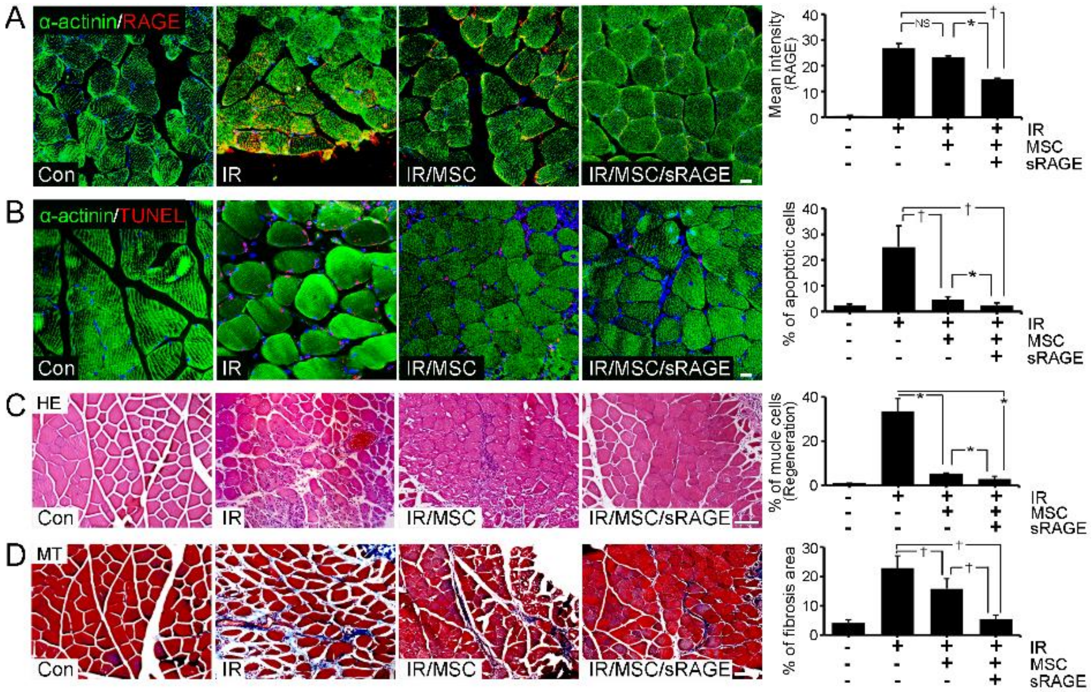

Figure S4. Reduced RAGE expression by sRAGE treatment in PIRI-CLI on 7 days

(A) The relative levels of RAGE (red) in skeletal muscle cells (α-actinin, green), after hBD-MSCs with (IR/MSC/sRAGE) or without sRAGE treatment (IR/MSC) in PIRI-CLI mice were evaluated by triple confocal microscopic analyses. (B) Apoptotic cells were illustrated by TUNEL (red) in skeletal muscle cells (green), after hBD-MSC treatment with or without sRAGE treatment. Scale bar = 20 ㎛ (C,D) The damaged skeletal muscles were measured by hematoxylin and eosin staining (HE) and fibrotic area was evaluated by Masson's trichrome (MT) staining in PIRI-CLI mice on 7 days. Scale bar = 50 ㎛. mean ± s.d. *, (P<0.05) and †, (P<0.01)

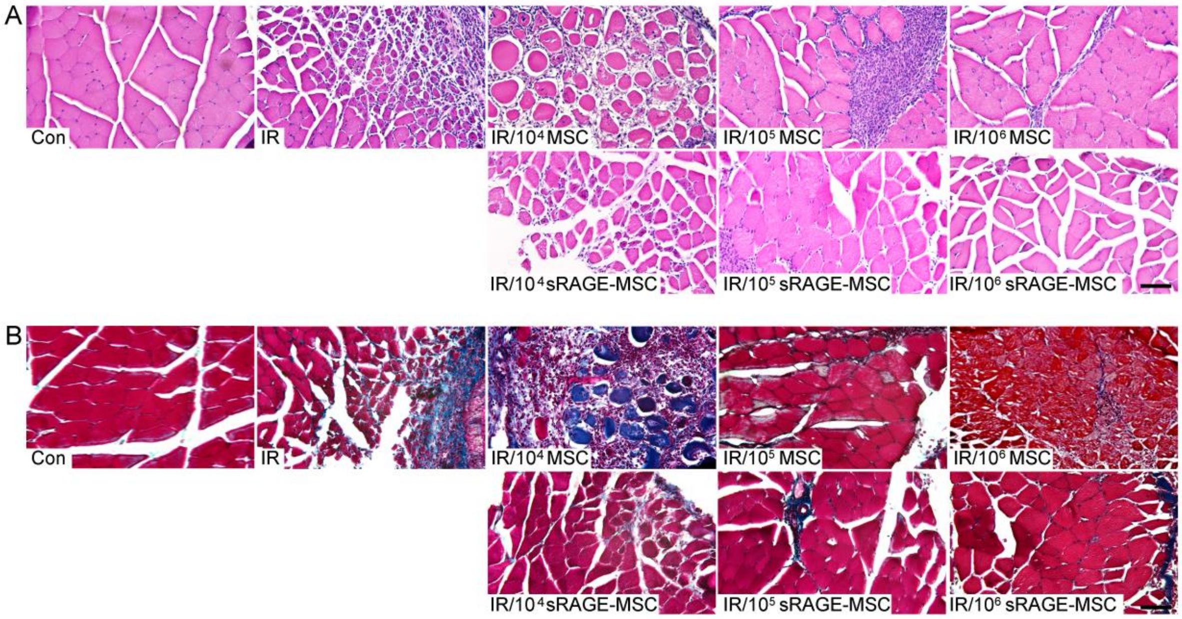

Figure S5. sRAGE improves the protective effect of hBD-MSC in low dosage cell number

(A, B) The damaged skeletal muscles were visualized by hematoxylin and eosin staining (HE) and fibrotic area was evaluated by Masson's trichrome (MT) staining in PIRI-CLI mice with the dose-dependent treatment of hBD-MSCs. Scale bar = 50 μm

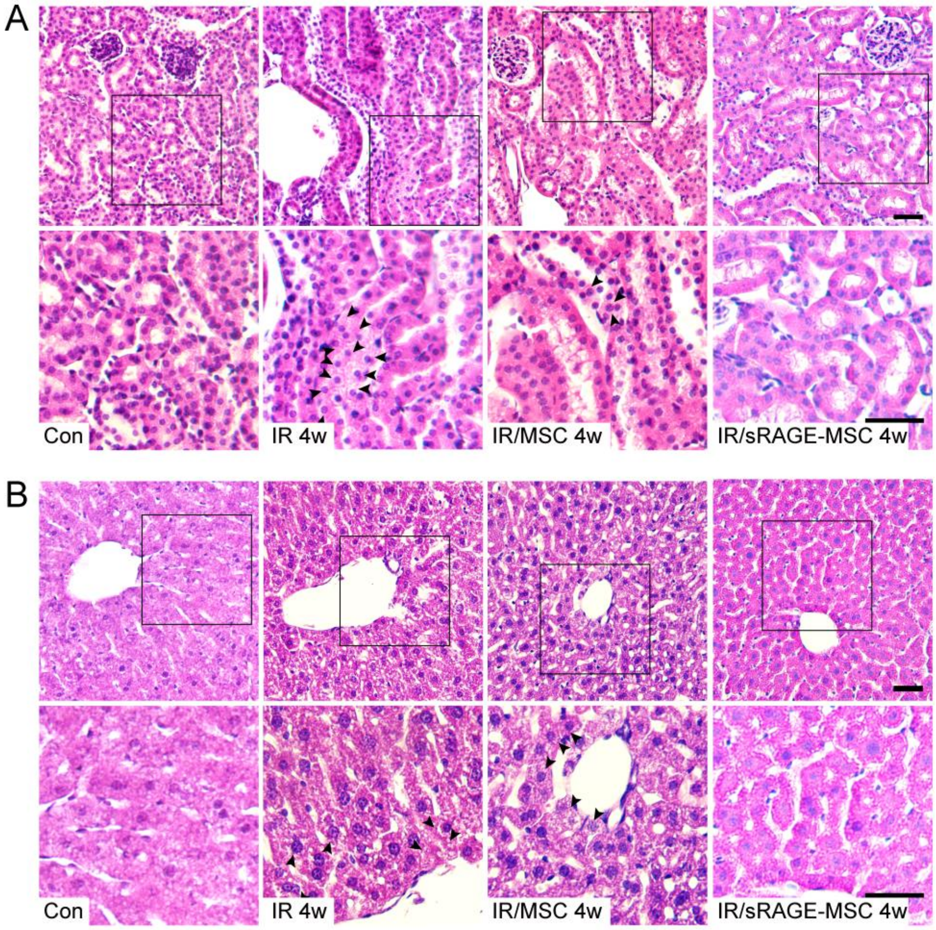

Figure S6. sRAGE-MSC has systemic protective effects on kidney and liver of PIRI-CLI model

The histological images show sRAGE-MSC has systemic protective effects in PIRI-CLI damaged kidney (A) and liver (B). Swollen tubular cells (arrow head) were observed in PIRI-CLI damaged kidney and hepatocellular necrosis (arrow head) in PIRI-CLI damaged liver. The sRAGE-MSC treatment showed the improved systemic protective effects in PIRI-CLI.

Supplemental Tables

Table S1. List of antibodies for immunoblotting and immunostaining

Antigen (host)	Company	Cat. No	Application	
			ICC/IHC	WB
β-actin (mouse)	Sigma-Aldrich	A5316	-	1:15,000
Erk1/2 (rabbit)	Cell signaling	9102S	-	1:1,000
pErk1/2 (rabbit)	Cell signaling	4377S	1:100	1:1,000
p38 (rabbit)	Cell signaling	9212L	-	1:1,000
pp38 (rabbit)	Cell signaling	9211S	1:100	1:1,000
SAPK/ JNK (rabbit)	Cell signaling	9252S	-	1:1,000
pSAPK, JNK (rabbit)	Cell signaling	9251S	1:100	1:1,000
Albumin (mouse)	Abcam	ab10241	1:100	-
AGE (rabbit)	Abcam	ab23722	1:200	-
Iba-1 (goat)	Abcam	ab5076	1:100	-
B7-2 (CD86, mouse)	Santa Cruz	sc-19617	1:100	1:100
CD206 (mouse)	Santa Cruz	sc-58987	1:100	1:100
α-actinin (mouse)	Sigma-Aldrich	A7732	1:800	-
CD44 (rabbit)	Abcam	Ab51037	1:50	
α-SMA (rabbit)	Abcam	Ab5694	1:200	
vWF (mouse)	Dako	A0082	1:200	
HIF-1α (mouse)	Abcam	ab463	1:200	-
HMGB1 (rabbit)	Abcam	ab18256	1:200	-
RAGE (goat)	Abcam	ab7764	1:400	1:4000
Peroxidase labeled anti-mouse IgG	Vector	PI2000	-	1:5000
Peroxidase labeled anti-rabbit IgG	Vector	PI1000	-	1:5000
Peroxidase labeled anti-goat IgG	Vector	PI9500	-	1:5000
Alexa Fluor 555 donkey anti rabbit IgG	Invitrogen	A31572	1:500	-
Alexa Fluor 633 goat anti rabbit IgG	Invitrogen	A21070	1:500	-
Alexa Fluor 555 donkey anti goat IgG	Invitrogen	A21432	1:500	-
Alexa Fluor 488 donkey anti mouse IgG	Invitrogen	A11001	1:500	-

Table S2. List of primers for Quantitative polymerase chain reaction (qPCR)

Gene		Primers
GAPDH	Forward	5'-CGT CTT CAC CAC CAT GGA AGA-3’
	Reverse	5'-CGG CCA TCA CGC CAC AGT TT-3’
CD86	Forward	5'-TCA GTG ATC GCC AAC TTC AG-3’
	Reverse	5'-TTA GGT TTC GGG TGA CCT TG-3’
CD206	Forward	5'-CCT CTG GTG AAC GGA ATG AT-3’
	Reverse	5'-CTT CCT TTG GTC AGC TTT GG-3’

Supplemental methods

PIRI-CLI animal group

The mice were anesthetized with a mixture of 2% xylazine hydrochloride (Rompun, 5-10 mg/kg; Bayer) and tiletamine/zolazepam (Zoletil 50, 25 mg/kg, Virbac Animal Health) through intra-peritoneal injection. After induction of anesthesia, surgical procedures were performed by following protocol. Briefly, longitudinal incision of left thigh skin was made. Proximal and distal ends of the femoral artery was ligated. Complete femoral artery occlusion was achieved and continued for 1 hrs. The ligation was removed and blood reperfusion was allowed into the ischemic muscle. After recovery of blood supply to the damaged muscles, the following 2 arms were established: 1) dose dependent hBD-MSC treatment: 1×10^4, 1×10^5 and 1×10^6 hBD-MSC or hBD-MSC with sRAGE, 2) validation of sRAGE protective effects: incubate for 7 days and 4 weeks after PIRI-CLI model generation.
